# Subclavian Artery Thrombosis in a COVID-19 Patient

**DOI:** 10.34172/aim.2022.78

**Published:** 2022-07-01

**Authors:** Ahmadreza Afshar, Ali Tabrizi, Ali Aidenlou

**Affiliations:** ^1^Department of Orthopedics, Imam Khomeini Hospital, Urmia University of Medical Sciences, Urmia, Iran

## Dear Editor,

 Although COVID-19 is primarily a respiratory infection, its complications are not necessarily limited to the respiratory tract. The extrapulmonary complications of COVID-19 may range from mild to very severe disorders. This case report describes a COVID-19 patient with coagulation abnormalities and thrombosis of the left subclavian artery that led to gangrene and trans-humeral amputation.

 A 61-year-old woman was admitted to the respiratory intensive care unit because of headache, malaise, cough, fever and respiratory difficulties. She had flu-like symptoms from one week before; however, her respiratory difficulties progressed. Her oxygen saturation was 85% on room air. Her real-time polymerase chain reaction (RT-PCR) test was positive for COVID-19. She did not have any known co-morbidities. The patient’s hemoglobin was 10.3 g/dL, white blood cell (WBC) was 14 000 per µL, erythrocyte sedimentation rate (ESR) was 45 mm/h, and C-reactive protein was 60 mg/L. She had not received vaccination for COVID-19 before.

**Figure 1:**
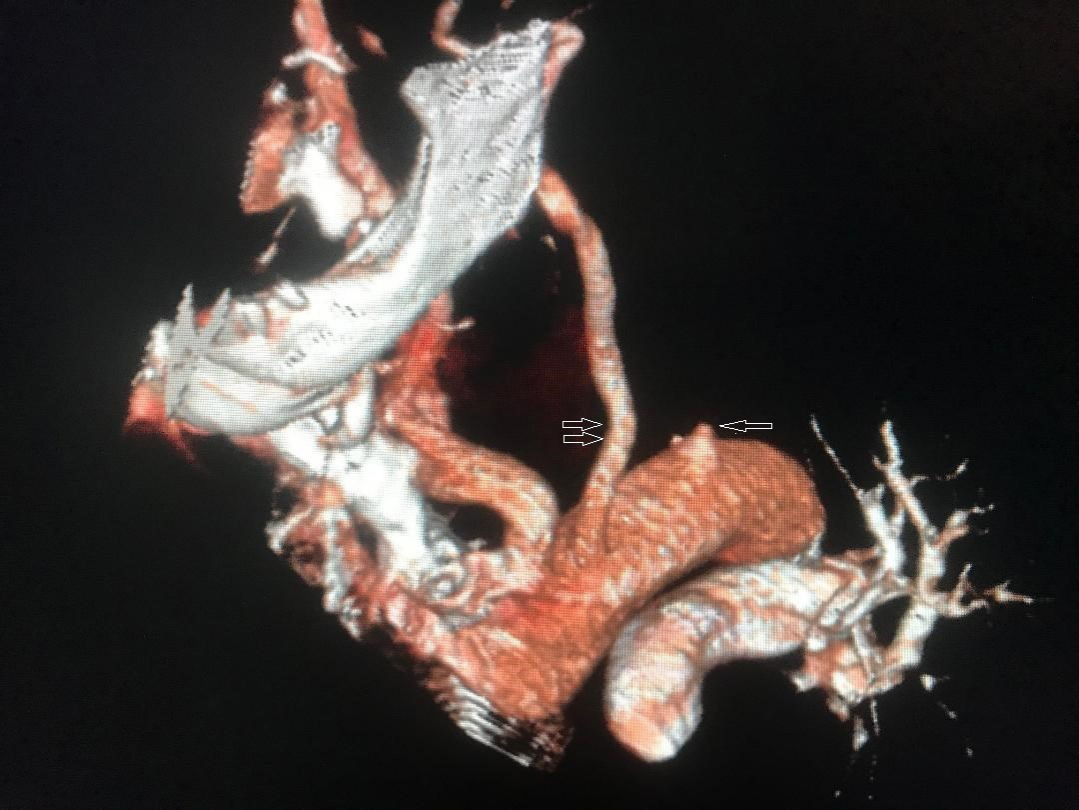
The single arrow on the CT angiogram demonstrates that the origin of the left subclavian artery is cut off. The origin of the left carotid artery is indicated with double arrows

 She was managed with oxygen through nasal catheter, steroids, remdesivir as an anti-viral medication, empirical antibiotics and anticoagulated with heparin. On the second day of her admission, her left hand circulation was lost and the hand became cool, mottled and painful. On examination, brachial, radial and ulnar pulses were absent. Doppler ultrasound confirmed occlusion of the three arteries. A CT angiogram demonstrated that the left subclavian artery was occluded at its origin from the aortic arch and there was no major distal perfusion in the left upper limb (Figure 1).

**Figure 2:**
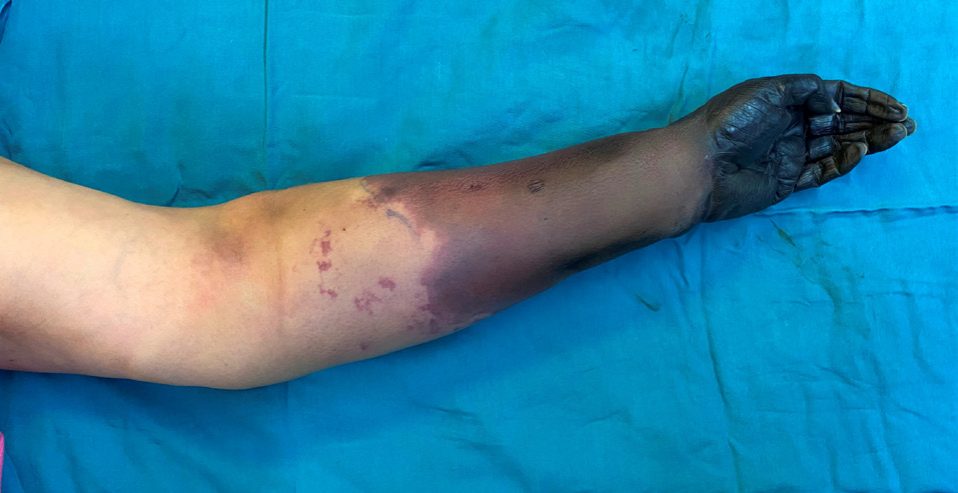
A 61-Year-Old Woman with COVID-19 and Compromised Circulation in the Left Upper Limb

 On day 7 of admission, she was referred to us because of an established and demarcated dry gangrene of the distal two third of left forearm and hand (Figure 2). Analysis of coagulation markers demonstrated that anticardiolipin antibody (IgM) was > 100 U/mL (reference: 12-18), antiphospholipid antibody (IgM) was 30 U/mL (reference: 12-18), lupus anticoagulant was at the upper normal limit of 44.8 seconds (reference: 31-44), fibrinogen was 646 mg/dL (reference: 200-400) and D-dimer was 2 mg/mL (reference level, 0.2 mg/mL). These analyses suggested hypercoagulopathy.

 Because of delayed referral, attempts to salvage the limb were ruled out; however, anticoagulation therapy with heparin was continued. An amputation through forearm was planned but postponed until a sufficient respiratory recovery from COVID-19 infection was achieved. On day 16 of admission, an amputation through forearm was performed; however, because of stump flaps necrosis, the below-elbow amputation was revised to trans-humeral amputation.

 There is a link between COVID-19 infection and complications of hypercoagulopathy.^1-3^ The virus provokes an inflammatory cascade that leads to a prothrombotic state resulting in micro- and macrovascular endothelial damage. Most reports have focused on venous thromboembolism while arterial thrombotic events, particularly thrombosis of the large named arteries, have received less attention.^1^ An acute limb ischemia may present in the form of chilblains, bullae, acral cyanosis, bruising, blood blisters, acute limb ischemia and life-threatening gangrene.^4-6^ Shao et alreported that an acute upper limb ischemia may be the first manifestation of COVID-19 infection.^7^

 In a review of 27 studies including 5 cohorts, 5 case series, and 17 case reports, Cheruiyot et alfound that arterial thrombosis had occurred in about 4% of critically ill COVID-19 patients which could affect multiple arteries. The anatomical distribution of arterial thrombotic events was 39% in limb arteries, 24% in cerebral arteries, 19% in large vessels including the aorta, common iliac, common carotid, and brachiocephalic trunk, 9% in coronary arteries, and 8% in superior mesenteric artery. The mortality rate in those patients was 20%.^8^

 Although COVID-19 is primarily a respiratory infection, its complications are not necessarily limited to the respiratory tract. The extrapulmonary complications of COVID-19 may range from mild to very severe disorders. This letter describes a COVID-19 patient with coagulation abnormalities and thrombosis of the left subclavian artery that led to gangrene and trans-humeral amputation.
